# A comparative analysis in monitoring 24-hour urinary copper in wilson disease: sampling on or off treatment?

**DOI:** 10.1186/s13023-025-03545-2

**Published:** 2025-01-21

**Authors:** Isabelle Mohr, Patrick Lamade, Christophe Weber, Viola Leidner, Sebastian Köhrer, Alexander Olkus, Matthias Lang, Andrea Langel, Patrischia Dankert, Melanie Greibich, Silke Wolf, Holger Zimmer, Patrick Michl, Aurélia Poujois, Karl Heinz Weiss, Uta Merle

**Affiliations:** 1 Internal Medicine IV, Department of Gastroenterology, University Hospital Heidelberg, INF 410, Heidelberg, 69120 Germany; 2 Internal Medicine III Department of Internal Medicine and Cardiology, University Hospital Heidelberg, Heidelberg, Germany; 3 Internal Medicine I, Department of Endocrinology, Diabetology, Metabolic Diseases and Clinical Chemistry, University Hospital Heidelberg, Heidelberg, Germany; 4 Department of Neurology, Rothschild Foundation Hospital, National Reference Center for Wilson Disease, Paris, France; 5 Internal Medicine, Salem Hospital Heidelberg, Heidelberg, Germany

**Keywords:** Wilson disease, Urinary copper, Exchangeable copper, Monitoring

## Abstract

**Background & Aim:**

Twenty-four-hour urinary copper excretion (24 h-UCE) is the standard diagnostic tool for dose adjustments in maintenance therapy in Wilson disease (WD) patients. Guidelines lack data if both variants of 24 h-UCE measurement (with or without 48 h of treatment interruption) are equally interpretable.

**Methods:**

Eighty-four patients with a confirmed diagnosis of WD treated with chelators (50% of patients with D-Penicillamine and 50% with trientine) and with pairwise 24-h-UCE values on-therapy and off-therapy were included in the analysis. Pairwise urinary sampling between October 2022 (T0) and a 12-month FU (T2) was compared, and exchangeable copper (CuEXC) was additionally measured at T0.

**Results:**

Among the 84 patients, 65% had predominant hepatic symptoms, the median age was 42 years, and 58% were female. At T0, patients were in the stable maintenance phase, with a median treatment duration of 21.9 years. The levels of the biochemical markers liver and copper metabolism remained stable over the 12-month observation period for all patients. 24 h-UCE off-therapy significantly decreased from T0 to T2 (*p* = 0.03), whereas no statistically significant differences were detected for 24 h-UCE after therapy. Both sampling methods did not correlate. CuEXC was significantly correlated with 24 h-UCE after 48 h of dose interruption (*p* = 0.018) but not with 24 h-UCE after therapy. A total of 46% of the 24 h-UCE value pairs were discordant, laying out the aimed therapeutic ranges given in current international guidelines.

**Conclusion:**

Off-therapy 24 h-UCE reflects the “free” copper pool more accurately than does urinary sampling. The study shows discordant results for both sampling methods in approximately half of the patients, revealing that interpretation of 24 h-UCE with respect to chelator-dosing decisions should be performed with caution.

**Supplementary Information:**

The online version contains supplementary material available at 10.1186/s13023-025-03545-2.

## Background

Wilson disease (WD) is an autosomal recessive disorder of copper metabolism resulting from various mutations in the ATP7B gene [1–3]. Excess copper primarily harms the liver and brain. WD can be treated with chelators (D-penicillamine (DPA), trientine) or zinc salts to achieve a negative copper balance [4–7]. Current guidelines [2, 8, 9] delineate two main stages of treatment: the initial copper reduction phase and long-term maintenance therapy. Regular follow-up examinations are recommended to monitor copper metabolism. In particular, 24 h-UCE delivers relevant data on the adequacy of dosing and necessary therapeutic adjustments [10–12]. As copper chelation with DPA and trientine results in increased urinary copper excretion, 24 h-UCE increases further when measured under copper chelation therapy. NCC (nonceruloplasmin-bound copper) has long been recommended for monitoring, reflecting so-called “free” copper. In untreated patients, NCC is usually high (> 15 µg/dL), whereas NCC normalization is in line with effective treatment. However, the NCC is calculated via a method that is dependent on ceruloplasmin. NCC is therefore considered a less reliable parameter than 24 h-UCE [13–15]. Instead of calculating the NCC, the “free” nonceruloplasmin-bound plasma copper fraction can be determined via ultrafiltration coupled with atomic absorption spectrometry. This labile copper fraction, which is termed “free” or exchangeable copper (CuEXC), is a new biochemical marker of copper overload independent of ceruloplasmin [16–18]. CuEXC (especially values > 2.08 µmol/l) is associated with extrahepatic involvement and its severity [19] and can also be applied in monitoring WD. Recently, published American guidelines recommend monitoring 24 h-UCE at least once per year [8]. For maintenance therapy, reference ranges for 24 h-UCE on chelator therapy are given in the American Association for the Study of Liver Diseases (AASLD) guidelines: 3–8 µmol/d under DPA therapy or within 2.4–8 µmol/d under trientine therapy [8, 9]. The measurement of 24 h-UCE after a 48 h-chelator-treatment-washout-phase is also possible and is recommended as an alternative to the AASLD guidelines but does not provide a specific range in which this off-chelator-24 h-UCE should be used [8]. The European Association for the Study of the Liver (EASL) guidelines of the year 2012 recommend aiming for a 24 h-UCE < 1,6 µmol/d off-therapy and a 24 h-UCE in the range of 3–8 µmol/d when measured on-therapy in the maintenance phase [2]. To date, no systematic comparison of both measurement options—on and off therapy—has been published; therefore, recent guidelines lack data if both measurements can be assumed to be equally valuable and equally interpretable. In our previously published retrospective monocentric study, the results of on-drug and 48 h-off-drug 24 h-UCE were compared, but these measurements were not performed pairwise in the same patient but rather in different patients and at different time points, rendering a comparison that is not ideal [15]. In this study, no significant decrease in 24 h-UCE was evident for longitudinal on-drug measurements. In contrast, a significant decrease in the 24 h-UCE rates in DPA-treated patients in the maintenance phase was demonstrated for off-drug UCE measurements. Our present study therefore aims to evaluate pairwise 24 h-UCE rates before and after therapy at the same time point and longitudinally over a 12-month period in a cohort of WD patients receiving maintenance therapy with chelating agents.

## Methods

### Study population

**Fig. 1:**
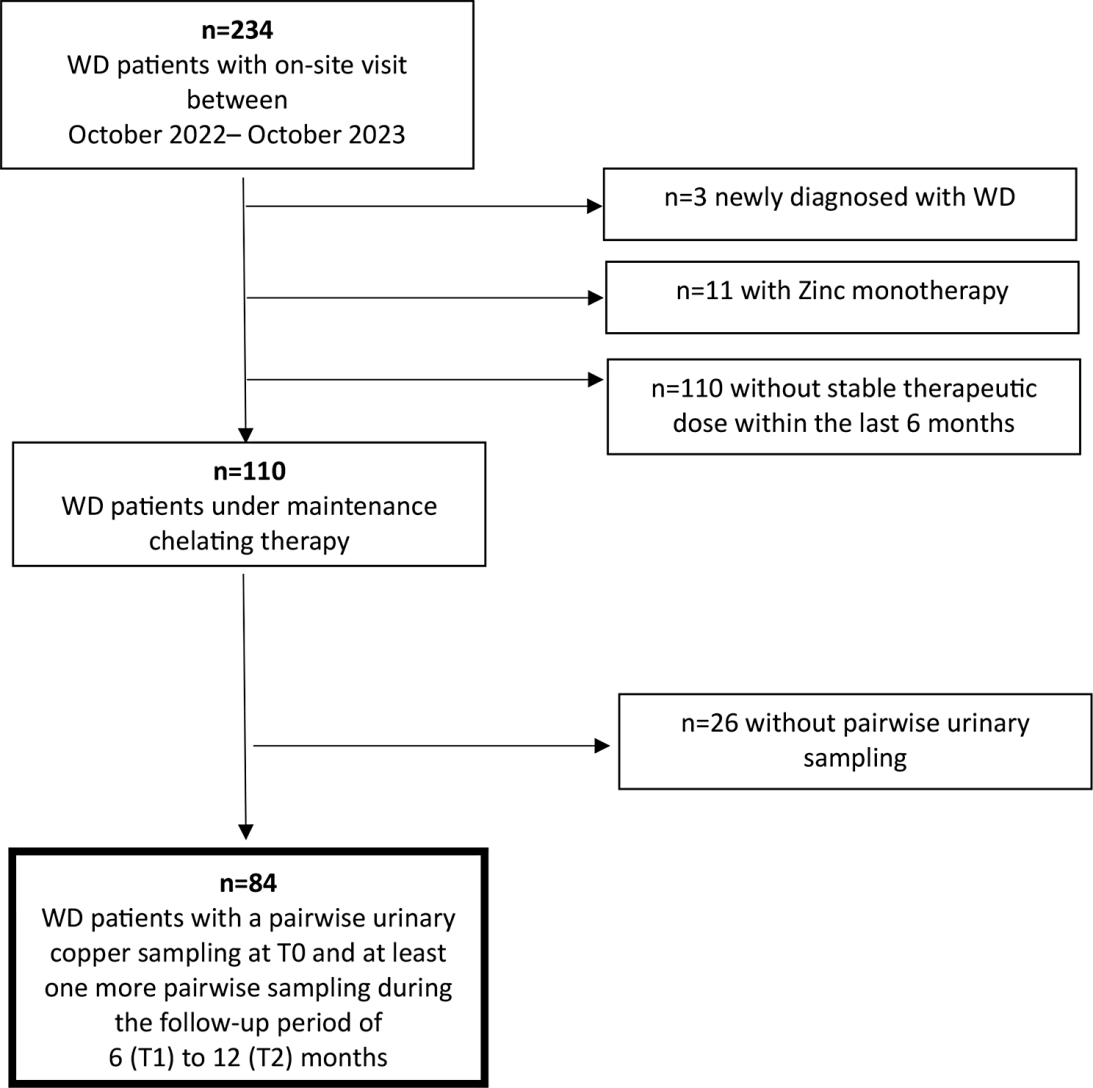
Study design The study cohort consists of 84 WD patients fulfilling the study criteria out of a total of 234. Patients newly diagnosed (*n* = 3), under zinc monotherapy (*n* = 11), or with dose adjustments during the prior 6 months (*n* = 110) were excluded. Patients without pairwise urinary sampling (*n* = 26) were also excluded from analysis

All patients with a confirmed diagnosis of WD (Leipzig score ≥ 4) who were treated from October 2022 to October 2023 at the Department of Gastroenterology and Hepatology of the University Hospital Heidelberg were retrospectively reviewed for inclusion in the study group (*n* = 234). Only patients receiving maintenance therapy and a stable chelator dose during the previous 6 months (signaling stable copper metabolism) were included. Patients who were newly diagnosed (*n* = 3), under zinc monotherapy (*n* = 11) or with dose adjustments during the prior 6 months (*n* = 110) were excluded. Patients fulfilling the inclusion criteria were instructed to collect two different 24-h urine samples beginning in October 2022, on and off therapy, and to perform pairwise urinary copper sampling in a standardized way. The first urinary sample was collected 14 days prior to the follow-up (FU) visit after 48 h of treatment interruption, with sampling still off therapy. The second 24-h urine sample was collected without treatment interruption the day prior to the FU visit (for the 24-h urine sampling method, see the supplemental methods Sect. 2). We included only patients with initial pairwise urinary copper sampling between October and December 2022 (T0) and at least one more pairwise sampling during the next 12 months—after 6 (T1) and/or 12 (T2) months. Patients fulfilling these criteria were retrospectively identified from October 2023. From a total of 234 WD patients treated at University Hospital Heidelberg, 84 patients were included in the data analysis (Fig. 1). The WD treatment was performed in accordance with current WD guidelines (AASLD, EASL) and drug labeling with trientine-dihydrochloride (TETA2 HCl) and tetrachloride (TETA4 HCl) for Germany, allowing for treatment with trientine only in patients who are intolerant to DPA treatment or with ineffective control of copper metabolism under DPA treatment. FUs were performed at the T0, T1 and T2 timepoints (inclusion and follow-up at 6 months and 12 months, respectively). Serum parameters of copper metabolism and hepatic function as well as noninvasive liver stiffness measurements (estimated by transient elastography) were collected. Serum ceruloplasmin was measured via a nephelometric (BNA2, Dade Behring) assay. Total serum copper was measured by atomic absorption spectrometry (AAS, VARIAN AA240FS). CuEXC was measured once in each patient at T0. For specific laboratory methods, refer to the supplemental methods Sect. 2. Patients were subclassified as having a primarily hepatic, neurological or mixed presentation.

### Statistical analysis

For statistical data analysis, the Excel database was exported into the statistical software IBM SPSS Statistics (Version 27.0, IBM, Chicago, IL, USA). To depict a primarily descriptive analysis of the study cohort, frequencies; measures of central tendency, such as the mean and median; and measures of dispersion, such as the minimum, maximum, and standard deviation, were used. Parametric methods were employed to investigate the temporal trends of individual laboratory parameters. A paired-samples t test was applied after confirming a normal distribution through the Shapiro‒Wilk test. The laboratory parameters during follow-up at time point T were statistically compared with the paired laboratory parameters at T0. For the statistical correlation of metric parameters, the Pearson correlation coefficient was used.

## Results

### Patient characteristics

Between October 2022 and December 2023, 84 WD patients (58% females) under maintenance chelating therapy with pairwise collection of 24 h urinary samples met the inclusion criteria and were examined. Among these patients, at initial diagnosis, 55 (65%) presented with predominant hepatic symptoms, 3 (4%) with predominant neurologic symptoms, and 26 (31%) with a mixed phenotype (Table 1).

**Table 1:** Patient baseline characteristics

Parameter	Patient characteristics
Number of patients	*n* = 84 (42% males, 58% females)
Demographics at baseline (median):	
age (years)	42 (range 18–77; SD 13.9)
weight (kg)	76 (range 48 to 115; SD 15.3)
height (cm)	176 (range 159 to 201; SD 0.1)
Median age at diagnosis	19 years (range 8 to 57; SD 12.0)
Treatment **at initial diagnosis** *concomitant therapy with zinc salts Median overall treatment duration of therapy at T0 (years) Treatment **at T0 (study Inclusion)**	D-Penicillamine; *n* = 74 (88.1%); (*n = 3) Trientine 2HCl; *n* = 5 (6%); (*n = 1) Trientine 4HCl: *n* = 1 (1.1%) Zinc monotherapy; *n* = 2 (2.4%) Tetrathiomolybdate *n* = 2 (2.4%) 21.9 (range 2.6 to 57.2; SD 14.0) D-Penicillamine; *n* = 42 (50%) Trientine 2HCl; *n* = 39 (46.4%) Trientine 4HCl; *n* = 3 (3.6%)
Predominant clinical presentation of WD at initial diagnosis	Hepatic; *n* = 55 (65%) Neurological; *n* = 3 (4%) Mixed; *n* = 26 (31%)
Liver cirrhosis	*n* = 17 (19.3%, all Child-Pugh A stage)

Table 1 depicts the baseline characteristics of the study cohort including demographics, median treatment duration and treatment regimen

Liver cirrhosis was present in 17 patients, all of whom had compensated stable Child‒Pugh A stage disease (median Child Pugh Score: 5.3 (SD 0.8) at T0 vs. 5.2 (SD 0.6) at T2). Stable liver cirrhosis in these cases was reflected in the absence of hepatic decompensation and a stable MELD (Model for End-stage Liver Disease) Score at T0 (7.9, SD 1.9) and after 12 months at T2 (7.7, SD 1.95).

The median CuEXC at T0 was 1.13 µmol/l (range 0.40–2,79; standard deviation (SD) 0.51). The median age at diagnosis of our study population was 19 years (range 8–57; SD 12.0). The median age of the patients at T0 was 42 years (range 18–77; SD 13.9). In patients with indications for chelating therapy, DPA was used as a first-line choice due to the admission requirements in Germany. Therefore, at initial diagnosis, most patients were started on DPA (88.1%). Since therapy initiation, initial treatment has been switched to trientine in many patients.Trientine (2 HCl or 4 HCl) was prescribed in patients intolerant to DPA treatment or with ineffective control of copper metabolism under DPA. At T0, the patient cohort had a median duration since the start of initial therapy of 21.9 years (range 2.6–57.2 years). At T0, 50% (*n* = 42) of patients were receiving therapy with DPA, and 50% (*n* = 42) were receiving therapy with trientine (TETA 2HCl or 4HCl).

### Key laboratory parameters during maintenance therapy

Monitoring WD patients under therapy aims to confirm treatment efficacy by demonstrating clinical and biochemical improvement or stability [8]. Within the study cohort, data on biochemical markers, with a focus on transaminases, liver synthesis parameters, blood count as an indirect sign of portal hypertension, and liver stiffness values (estimated by transient elastography), revealed a stable WD condition in all included patients. A paired-samples t test was applied for all laboratory parameters, and the FU data were compared with the laboratory parameters at T0. None of these values differed significantly from the values at T0 (Table 2). When the dosage per day and per kilogram body weight were compared, the therapeutic dose was nearly the same at T0 and at both FU time points over the 12-month observation period in patients under both DPA and trientine (Supplemental Tables 1a and 1b).

**Table 2:** Longitudinal descriptive statistics for key laboratory parameters and transient elastography

Parameter	T0		T1		T2		T0 vs. T2
	n	Median (range; SD)	n*^1^	Median (range; SD)	n*^1^	Median (range; SD)	p-value*^2^
AST [U/L]	84	28 (12–288; 33)	82	36 (12–83; 13)	68	27 (6–56; 12)	0.05
ALT [U/L]	84	33(10–121; 25)	82	43 (14–198; 32)	68	38 (15–107; 26)	0.98
GGT [U/L]	84	26 (7-432; 59)	82	29 (8-366; 47)	68	29 (12–271; 39)	0.61
AP [U/L]	84	83 (42–392; 62)	82	87 (28–180; 29)	68	87 (34–182; 27)	< 0.78
Bilirubin [U/L]	84	0.7 (0.2–5.9; 0.8)	82	0.7 (0.3–4.5; 0.6)	68	0.7 (0.3–2.4; 0.4)	0.22
INR	84	1.0 (0.9–1.8; 0.1)	82	1.1 (0.8–1.4; 0.1)	68	1.0 (0.9–1.4; 0.1)	0.22
WBC [/nl]	84	5.9 (2.6–10.4; 1.6)	82	5.4 (2.4–10.9; 1.6)	68	5.2 (0.5–10.6; 1.7)	0.17
Thrombocytes [/nl]	84	207 (43–411; 74)	82	208 (42–346; 66)	68	214 (16–330; 65)	0.51
24 h Urinary copper excretion [µmol/d] after 48 h dose interruption	84	1.6 (0.2–5.2; 1.3)	67	1.7 (0.2–8.6; 1.3)	45	1.5 (0.4-4.0; 0.9)	**0.03**
24 h Urinary copper excretion [µmol/d] without dose interruption	84	6.5 (0.4–34.5; 6.0)	67	8.6 (1.5–26.2; 6.1)	45	7.7 (2.0 -24.2; 4.6)	0.36
Serum copper [µmol/L]	78	6.4 (1.3–19.9; 4.1)	65	6.7 (0.9–16.3; 6.1)	68	6.1 (0.9–16.3; 3.8)	0.36
Coeruloplasmin [g/L]	84	0.2 (0.03-8.0; 0.9)	63	0.1 (0.02–0.3; 0.07)	66	0.1 (0.06–0.3; 0.07)	0.34
NCC [g/L] (calculated)	84	1.9 (0.4–7.1; 1.6)	37	1.6 (0.1–7.3; 1.3)	35	1.0 (0.1–3.5; 1.0)	0.46
CuEXC [µmol/L]	84	1.13 (0.40–2.79; 0.51)					
Transient elastography Stiffness [kPa] CAP [dB/m]	59 59	6.1 (2.7–38; 4.8) 240 (139–399; 51)	58 58	6.1 (3.5–44; 6.0) 230 (126–366; 54)	57 57	6.0 (3.5–33; 4.8) 233 (177–371; 56)	0.38 0.96

*^1^ as not all patients were seen at both, T1 and T2 follow-up timepoints, data was not available for all patients

*^2^ p-value was obtained using paired sample t-test, only paired values were included in the statistical calculation

Legend

AST[U/L] = aspartate aminotransferase; ALT [U/lL] = alanine aminotransferase; GGT [U/L] = amma-glutamyltransferase; AP[U/L] = alkaline phosphatase; INR = international normalized ratio; WBC = white blood cell count; NCC = nonceruloplasmin bound copper; CuEXC = exchangeable copper; CAP = Controlled Attenuation Parameter; SD = standard deviation, n = number

Table 2 depicts longitudinal development of key laboratory parameters of liver function parameters, transient elastography and copper metabolism at T0, T1 and T2

### Analysis of pairwise 24 h urinary copper excretion on and off chelator therapy and correlation with other copper metabolism parameters

**Fig. 2:**
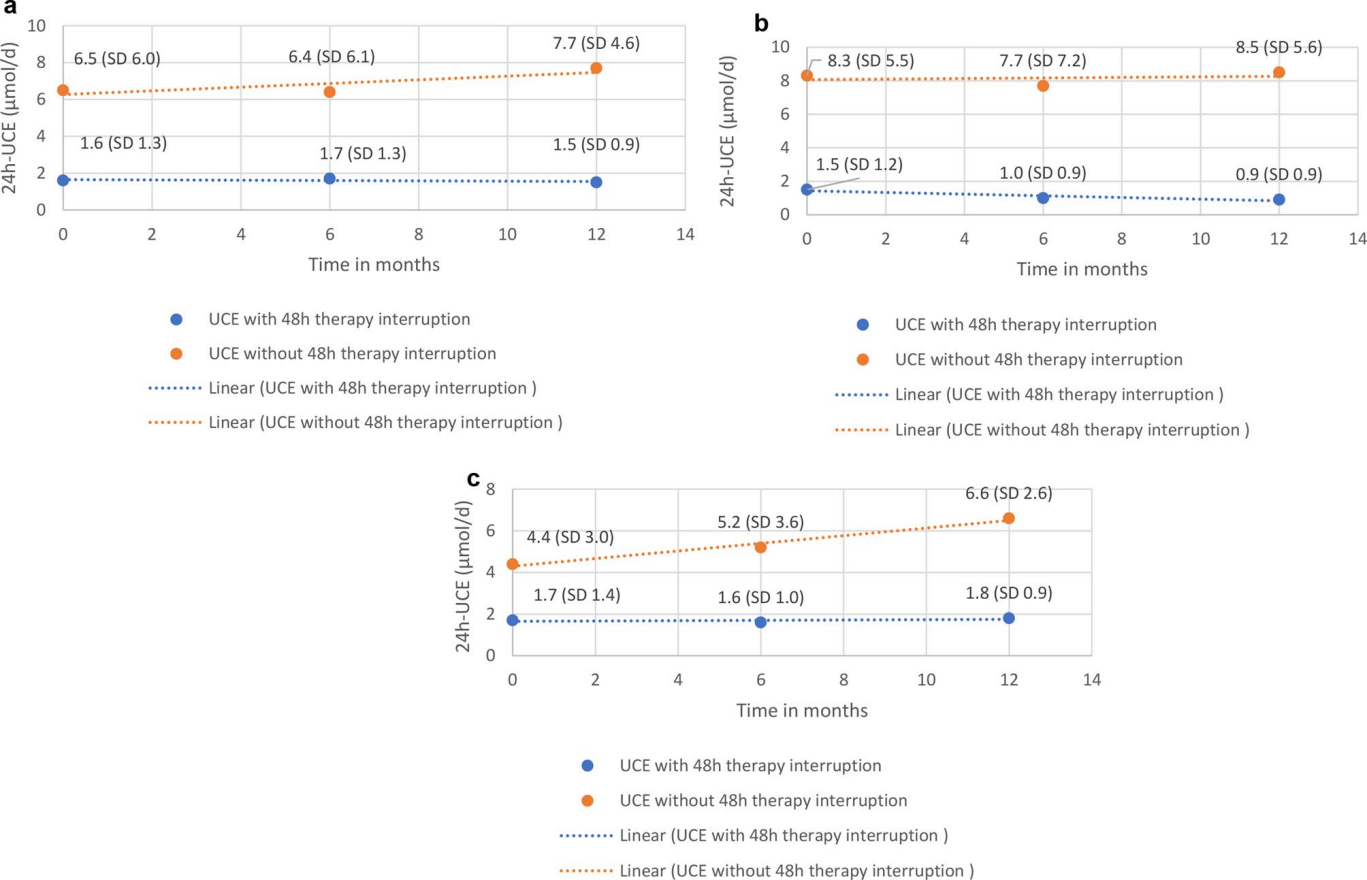
(**a**) Longitudinal development of 24 h-UCE overall. Longitudinal development of median 24 h-UCE values (and their respective standard deviations) in the overall cohort are depicted at T0, T1 and T2. (**b**) Longitudinal development of 24 h-UCE under trientine. Longitudinal development of median 24 h-UCE values (and their respective standard deviations) of WD patients under trientine therapy are depicted at T0, T1 and T2. (**c**) Longitudinal development of 24 h-UCE under D-Penicillamine. Longitudinal development of median 24 h-UCE values (and their respective standard deviations) of WD patients under D-Penicillamine therapy are depicted at T0, T1 and T2

The mean 24 h-UCE values measured during therapy at T0, T1, and T2 were 6.5 µmol/d (SD 6.0), 6.4 µmol/d (SD 6.1), and 7.7 µmol/d (SD 4.6), respectively, and the mean 24 h-UCE values after therapy were 1.6 µmol/d (SD 1.3), 1.7 (SD 1.3) µmol/d and 1.5 µmol/d (SD 0.9), respectively. When the ranges and standard deviations of both 24 h-UCE sampling methods were compared, the values measured after 48 h of dose interruption showed considerably narrower ranges and smaller standard deviations (Fig. 2a).

The mean 24-h UCE values measured during DPA therapy were 8.3 µmol/d, 7.7 µmol/d and 8.5 µmol/d, respectively, and the mean 24 h-UCE values measured during off-DPA therapy were 1.5 µmol/d, 1.0 µmol/d and 0.9 µmol/d, respectively. The difference in the mean 24 h-UCE between T2 and T0 did not reach statistical significance, neither for on-treatment UCEs nor for off-treatment UCEs. When the ranges and standard deviations of both sampling methods were compared, the values measured after 48 h of dose interruption presented considerably narrower ranges and standard deviations (Fig. 2b).

The mean 24 h-UCE values measured during trientine therapy were 4.4 µmol/d, 5.2 µmol/d and 6.6 µmol/d, respectively, and the mean 24 h-UCE values measured during off-trientine therapy were 1.7 µmol/d, 1.6 µmol/d and 1.8 µmol/d, respectively. The comparison of the mean UCEs at T2 and T0 did not reach statistical significance for either the on-treatment UCEs or the off-treatment UCEs. When the ranges and standard deviations of both sampling methods for the UCEs were compared, the UCEs measured after 48 h of dose interruption presented considerably narrower ranges and standard deviations (Fig. 2c).

**Fig. 3:**
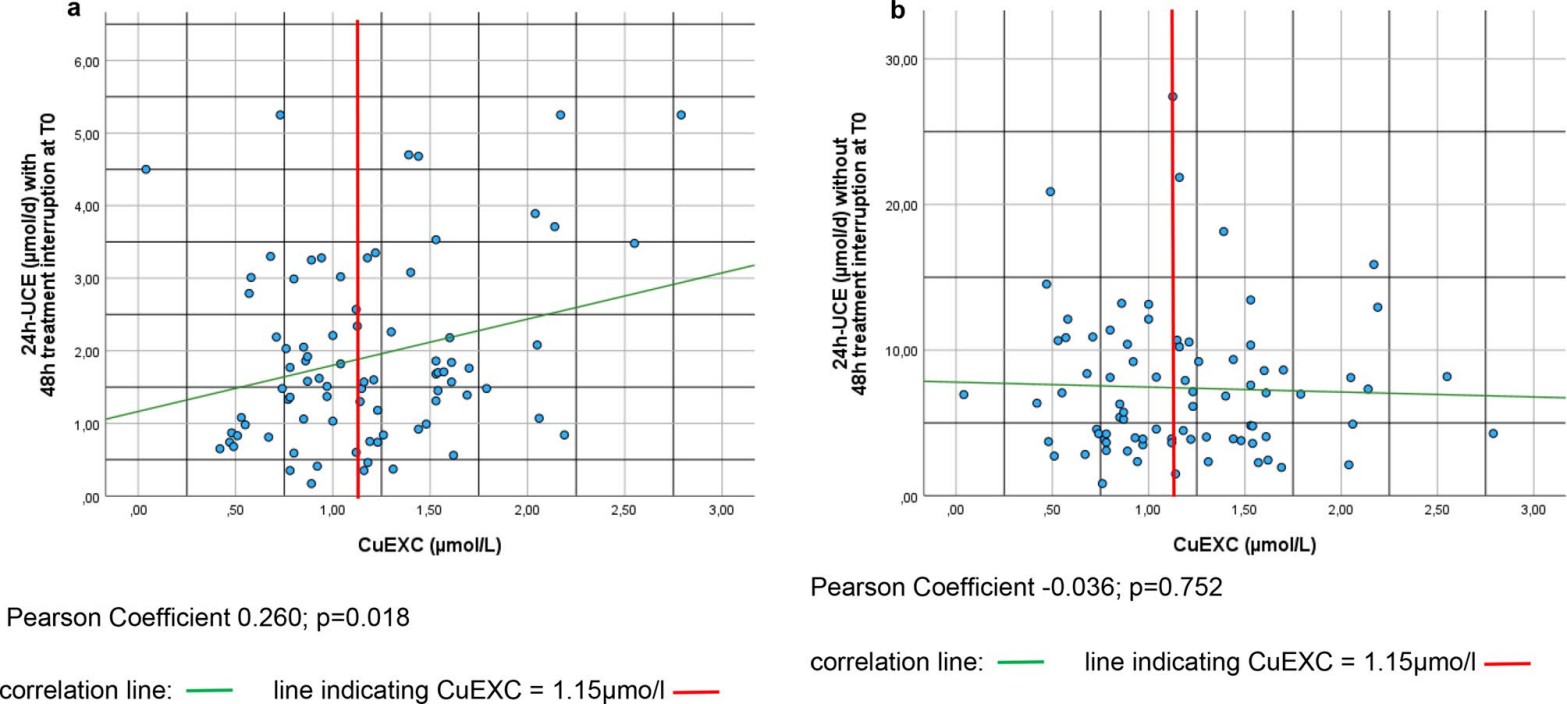
Correlation of CuEXC and 24 h-UCE. Correlation of 24 h-UCE on-therapy (**a**) and correlation of 24 h-UCE off-therapy (**b**) with CuEXC are depicted in Fig. 3. Correlation line is drawn in green whereas CuEXC cut-off value is drawn in red

Analysis of the longitudinal values of 24 h-UCE collected off therapy revealed lower median values at T1 and T2 than at T0, with a significantly lower 24 h-UCE at T2 than at T0 (1.5 µmol/d at T2 versus 1.6 µmol/d at T0; *p* = 0.03). In contrast, 24 h-UCE was not significantly different between T2 and T0 (7.7 µmol/d versus 6.5 µmol/d; *p* = 0.36; Fig. 2a). Longitudinal trajectories of on- and off-therapy 24 h-UCE values showed considerable variation between the three timepoints for both modes of measurement (Supplemental Fig. 1a and 1b). Analysis of the correlation of on- and off-medication 24 h-UCE values revealed no significant correlation between these pairwise measurements (Supplemental Table 2). CuEXC should provide a reliable estimation of the “free” copper fraction in the serum. To better understand the differences in the results of 24 h-UCE measurements before and after therapy, CuEXC was determined and correlated with these measurements. Off-therapy 24 h-UCE and CuEXC were weakly but significantly correlated, with a correlation coefficient of 0.260 (Fig. 3a), whereas on-therapy 24 h-UCE values were not significantly correlated with the CuEXC values (Fig. 3b).

Other parameters of copper metabolism (total serum copper, serum ceruloplasmin, and calculated NCC) were not significantly different between T0 and T2 (Table 2). Correlation analysis of the NCC at each timepoint with the corresponding UCE values on- and off-therapy also revealed no significant differences, neither for 24 h-UCE on treatment nor for 24 h-UCE off treatment (Supplemental Table 2a and Supplemental Sect. 1).

### Comparison of pairwise 24 h-UCE values with respect to their congruent or discordant implications

**Fig. 4:**
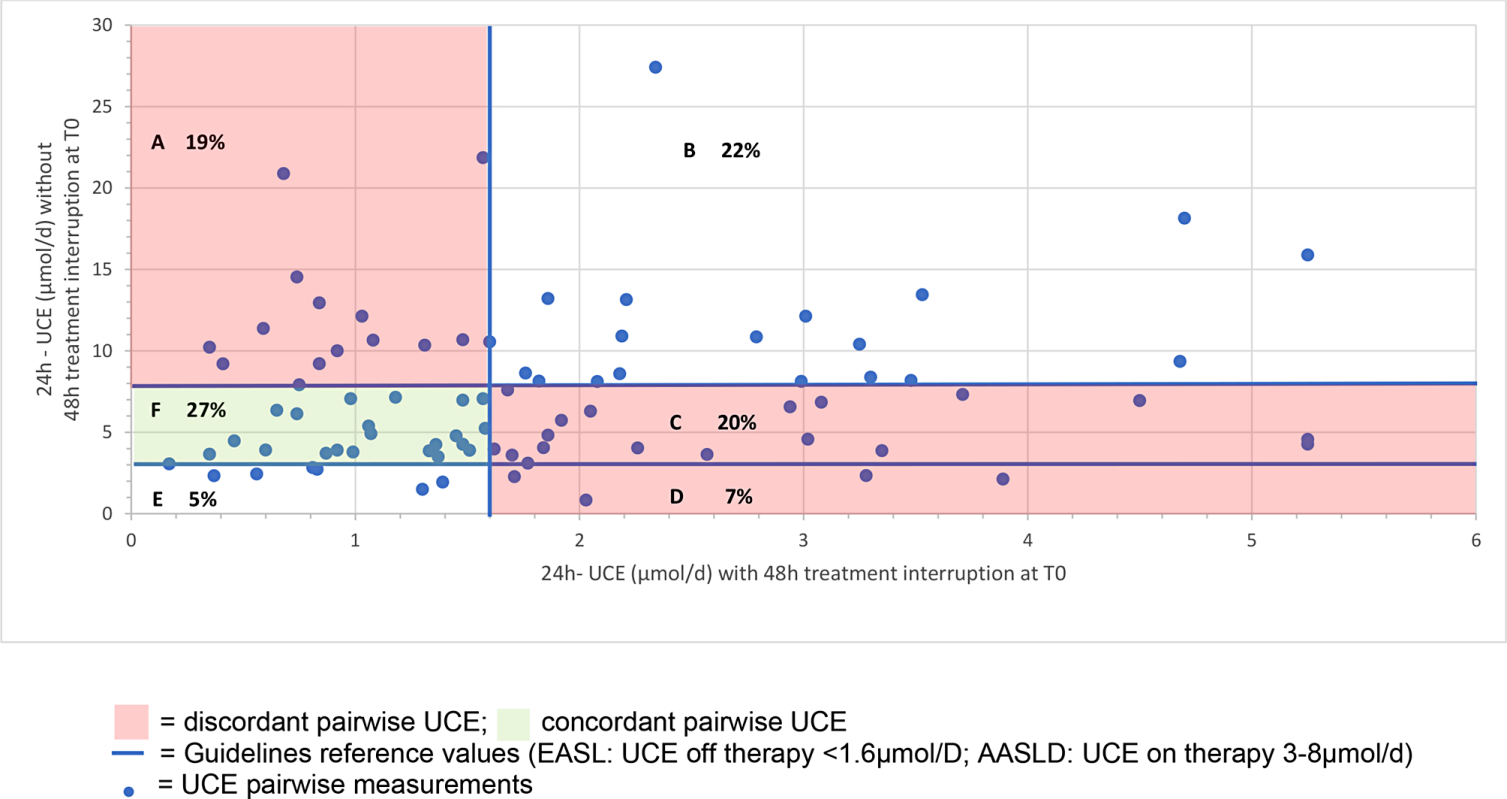
Scatterplot of T0 measurements of 24 h UCE with and without treatment interruption. The Scatterplot shows 24 h-UCE of T0 measurements with and without treatment interruption. Guideline reference values are drawn with blue lines (EASL off therapy < 1.6µmol/D; AASLD on therapy 3–8µmol/d). Green box shows concordant measurements, whereas red boxes reveal discordant values. Additionally, percentage of measurement of each box (**A** to **F**) is given in %

The longitudinal off-treatment 24 h-UCE values were within the targeted range of < 1.6 µmol/d in 51% (T0), 66% (T1) and 56% (T2) of patients. For on-therapy 24 h-UCE measurements, the results were within the targeted range of 3–8 µmol/d in 47% (T0), 61% (T1) and 47% (T2) of patients (Fig. 4, Supplemental Fig. 2a and 2b).

Although the proportions of both 24 h-UCE sampling methods within the therapeutic goal were within a similar range and ranged between 47% and 66%, the paired values were rarely within the target range. The concordance between the two parameters was as low as 27% at T0 (Fig. 4, field F). This similarly held true for T1 (34%) and T2 (22%) (Supplemental Fig. 2a and 2b). The pairwise 24 h-UCE values were also concordantly above the therapeutic target range of 22% (Fig. 4, field B). In 46% of pairwise measurements, the results were discordant, with 19% of value pairs above the therapeutic target value for on-therapy samples but within the therapeutic target range for off-therapy samples (Fig. 4, field A), 20% of value pairs in the therapeutic aim for on-therapy values but above the therapeutic aim for off-therapy values (Fig. 4, field E), and 7% of value pairs below the therapeutic aim for on-therapy values but above the therapeutic aim for off-therapy values (Fig. 4, field D). Therefore, decision-making on the basis of 24 h-UCE results would lead to a relevant percentage of patients making different decisions depending on the 24 h-UCE method chosen. 5% of pairwise values were below the therapeutic threshold of 3 µmol/d for on-therapy measurements and, in parallel, below 1.6 µmol/d for off-therapy 24 h-UCE values, with a lower threshold for the therapeutic goal for off-therapy measurements being not clearly defined (Fig. 4, field E). Overtreatment in an adherent patient can be assumed if 24 h-UCE measured on therapy is less than < 1.6 µmol/d.

## Discussion

WD patients should be monitored regularly under treatment to assess the response to treatment, to identify potential side effects and to ensure adherence to the prescribed medication. In addition to clinical and standard laboratory tests, copper metabolism parameters should be measured for treatment monitoring. For long-term treatment, sustained clinical and laboratory improvement is the most crucial indicator of treatment efficacy. Compared with other studies, the patient cohort mirrors a typical cross-sectional maintenance group of WD patients with a typical distribution of clinical presentations in line with classical WD characteristics, particularly hepatic and neurological manifestations [3, 17, 20–22]. We included only patients under maintenance therapy (50% of patients with DPA and 50% with trientine) with stable dosing in our study to maximize the longitudinal comparability and stability of 24 h-UCE. Statistical analysis revealed no significant differences between the longitudinal measurements of biochemical markers with a focus on transaminases, liver synthesis parameters, or copper metabolism (NCC, serum copper) as a sign of sufficient copper control [16, 23]. Appropriate monitoring of copper balance is essential to assess treatment efficacy and to avoid undertreatment (insufficient medication dosage or nonadherence) and overtreatment, which may result in copper deficiency. Currently, WD treatment monitoring lacks reliable and fully validated bioanalytical methods. As recommended in international guidelines, 24 h-UCE can be measured on or after a short pausing of chelator therapy. In the initial phase after the start of medical therapy for WD, 24 h-UCE values are highest and decrease over time during maintenance therapy [24–26]. On the basis of international guideline recommendations, 24 h-UCE should be between 3 and 8 µmol/d on maintenance therapy with chelators. 24 h-UCE values above 8 µmol/d in treated patients in the maintenance phase suggest insufficient drug action (nonadherence to medication, inadvertently low dosing or too low an absorbed chelator dose) or excessive copper dietary copper uptake [2, 9, 24, 27–29]. On-chelator therapy resulting in 24 h-UCE < 1.6 µmol/d may signal overtreatment or nonadherence. With overtreatment, CuEXC and 24 h-UCE should be concordantly low, whereas nonadherence would result in low 24 h-UCE but increased CuEXC. Moreover, CuEXC is often utilized for therapy monitoring, especially in France but also in other countries (Spain, Denmark, India, etc.) [30, 31], but has not been recommended for clinical practice in any other country. However, CuEXC is an accurate diagnostic tool for therapy monitoring in WD patients. Ngawanou et al. reported a significant decrease in CuEXC and 24-hour UCE levels during the first year of follow-up in children with WD. In another study focusing on adherence to treatment in WD patients, the levels of CuEXC were significantly higher in the group of patients with low adherence than in the group combining medium and high adherence [32].Additionally, the dynamics of both biomarkers were similar throughout the follow-up, demonstrating their usefulness in clinical practice for monitoring WD [33]. The European WD guidelines of 2012 [2] recommend the cessation of chelator therapy two days before determination of 24 h-UCE but mention measurement of therapy as a potential alternative. European guidelines recommend 24 h-UCE < 1,6 µmol/d after 48 h of therapy interruption and 24 h-UCE of 3–8 µmol/d during treatment [2]. The current American guidelines recommend measurements during treatment and mention off-therapy 24 h-UCE determination as a potential alternative [8]. The American guidelines recommend a range of 24 h-UCE of 3–8 µmol/d for DPA and 2.4–8 µmol/d for trientine, without differentiating between the 24 h-urinary sampling methods [8, 9]. Additionally, the American guidelines state that measuring UCEs after a 48 h washout period off drugs may be informative for assessing the adequacy of treatment [8]. In theory, off-treatment 24 h-UCE measurement in the maintenance phase should result in normalized 24 h-UCE. In summary, although both modes of UCE determination are possible and recommended as alternatives in current guidelines, to date, it is unclear if they are equally reliable. Studies comparing pairwise 24-h UCE measurements with longitudinal follow-up data are lacking. Pfeiffenberger et al. studied UCE values during and after treatment (d-penicillamine, trientine and zinc) but not via pairwise determination. They concluded that on-treatment 24 h-UCE is influenced mainly by the chelating dose, whereas off-treatment 24 h-UCE is a function of free copper [15]. In all the treatment groups, the UCE rates decreased during long-term observation (until 60 months), with considerable interindividual variation showing a greater effect for DPA than for trientine. However, median off-therapy UCE rates decreased more pronouncedly than did values taken on-therapy [15]. In our present study, longitudinal 24 h-UCE decreased significantly only when measured after a 48-h dose interruption from T0 to T2. In comparison, 24 h-UCE did not significantly change after 12 months. Additionally, both sampling methods did not correlate with each other. Chelating therapy aims to reduce the pool of toxic “free” copper [4, 25], and CuEXC is assumed to reflect this pool. In our study, CuEXC was significantly positively correlated with off-therapy 24 h-UCE values, whereas there was no significant correlation between CuEXC and on-therapy 24 h-UCE values. As CuEXC reflects unbound copper, we hypothesize that the 24 h-UCE measured off-therapy reflects the toxic “free” copper more accurately than the on-therapy UCE measurement does. This is in line with the findings of Pfeiffenberger et al. [15]. In addition to CuEXC, other laboratory methods that measure “free” copper directly have been developed. During the CHELATE trial, a liquid chromatography assay was developed using inductively coupled plasma‒mass spectrometry (ICP-MS) [34] for determining NCC (NCC-MS). Paired samples for NCC-MS and 24 h-UCE on therapy at the day of enrollment vs. 24 weeks postrandomization were compared [35, 36]. Interestingly, 24 h-UCE values measured during therapy were within the target range of 3–8 µmol/d in only 41% of the patients at the beginning and in 37% of the patients 24 weeks after randomization. Concordance between both parameters (NCC-MS and 24 h-UCE measured during therapy) was reached in only 35% of the values before randomization and in 33% of the values 24 weeks after randomization. Additionally, NCC-MS revealed less intraindividual variability than 24 h-UCE did during therapy [35]. A current study by Kirk et al. [37] examined the intestinal absorption of ^64^Cu and the effects of absorption under DPA and trientine treatment in healthy volunteers by using positron emission tomography (PET CT). The main finding was that treatment with trientine reduced hepatic ^64^Cu content very effectively (> 50%) within 15 h by blocking intestinal copper absorption and therefore increasing faecal elimination. The same was not observed after DPA treatment, strongly suggesting that DPA does not reduce the intestinal copper uptake by at least 50% but moreover increases renal copper excretion. This may explain why trientine and DPA are equally effective although urinary copper excretion is lower with trientine. Taken these relatively new findings from the ^64^Cu PET study into account, it seems plausible, that therapeutic targets for 24 h-UCE might be different for both drugs. Comparing the 24 h-UCE results under continued trientine (Fig. 2b) with those for under continued DPA treatment (Fig. 2c), the results for 24 h-UCE under trientine are lower and those under DPA higher, reflecting different copper urinary copper elimination putatively due to different effects on intestinal copper absorption, which is in line with the findings from the ^64^Cu PET Study [37]. After 48 h of therapy cessation, these early absorption effects with regard to the pharmacokinetic profile of both substances are levelled, strengthening our findings, that 24 h-UCE after therapy cessation is more consistent. These findings support our hypothesis that 24 h-UCE measured during therapy does not precisely reflect copper metabolism. Nevertheless, there is a slow elimination phase lasting 4 to 6 days for DPA, which suggests that there is a ‘slow pool of the drug reversibly bound to tissues’ especially when long term treatment of DPA is discontinued [38]. This needs to mentioned as a potential confounder for the 24 h- UCE off-therapy. 24 h-UCE measured on therapy appears to deliver information mainly whether recent treatment dosage is sufficient or whether there is overtreatment. In contrast, 24 h-UCE off-therapy appears to be the more suitable parameter to examine the “free” copper amount in plasma and therefore correlates better with CuEXC.To better understand the concordance or of 24 h-UCE measurements on- and off-therapy, we compared both urinary sampling methods on the basis of the recommendations of international guidelines. Over the observational period of 12 months, the therapeutic goal of UCE < 1.6 µmol/d after 48 h of therapy interruption was reached by 51–66% of patients. In contrast, the therapeutic target range of 3–8 µmol/d was reached by 47–61% of patients sampling on-treatment. When the ranges and standard deviations of both sampling methods were compared, UCE with 48 h of dose interruption revealed considerably narrower interindividual ranges and less statistical variance (Fig. 2). Overall, the values seemed to fluctuate less within one individual when sampling after 48 h of treatment discontinuation. These findings are in line with the results of the CHELATE trial: 24 h-UCE therapy resulted in greater intraindividual variability than did NCC-MS [35]. The correlation between the two sampling methods was rather weak in our study cohort. Pairwise comparisons of on- and off-treatment samples at T0 revealed concordant results in only 49% of patients: in 22%, both values were outside the therapeutic target range, and in 27% of patients, both sampling methods yielded values within the target range (Fig. 4, B and F). At T1 and T2, the results were even more inconsistent (Supplemental Fig. 2). 47% of the pairwise results were discordant and would have indicated different treatment decisions on the basis of the underlying sampling method (Fig. 4, A, C and D). Notably, the clinical indications derived from 24 h-UCE values are not identical in almost half of the patients. On the basis of the 24 h-UCE results, dose escalation was recommended for 41% of patients at T0 (Fig. 4, A and B). On the basis of the 24 h-UCE results, dose escalation was recommended for only a fraction of these patients (22%) (Fig. 4, B). Conversely, in 20% of the patients, off-treatment sampling would indicate dosage adjustments, whereas on-treatment sampling would indicate the correct dosage (Fig. 4, C). Overall, value pairs might provide the best tool for characterizing a patient’s current copper metabolism status. A patient in the maintenance phase with an off-therapy 24 h-UCE within the therapeutic range and an on-therapy 24 h-UCE above the therapeutic range could have increased dietary copper intake, leading to high 24 h-UCE. In contrast, a nonadherent patient could be identified by low 24 h-UCE on-therapy but increased 24 h-UCE off-treatment. For all patients with discordant values for sampling on- and off-treatment, excessive copper uptake via nutrition and nonadherence to therapy should be evaluated thoroughly before dosage adjustments. CuEXC testing might provide more plausible insights when in doubt. For the first time, this study provides relevant data regarding 24 h-UCE sampling methods in a real-world cohort of stable WD patients receiving maintenance therapy with chelating agents. The 24 h-UCE values before and after treatment were compared in a pairwise manner over a 12-month observational period. Interpretation of the results might be limited by the sample size of the cohort due to the inclusion criteria (receiving maintenance therapy and a stable chelator dose during the previous 6 months), raising some caution concerning the statistical power of this retrospective, monocentric study. Real-world data might not reflect unintentional sampling errors of patients or study data. However, the real-world study characteristics might be advantageous since potential confounders such as patient variables (e.g., nonadherence) might be realistically depicted. The presented data raise the concern of whether urinary sampling with and without therapy interruption is equally interpretable, showing that the use of both sampling methods can result in incongruent dose adjustment recommendations under maintenance therapy in nearly 50% of patients. Further randomized studies with longer observational periods and patients in different therapeutic stages (decoppering vs. maintenance phase) and under different therapy regimens (chelating agents vs. zinc) are needed to further validate our findings.

## Conclusion

During the maintenance phase of chelation, off-therapy 24 h-UCE seemed to reflect the “free” toxic copper pool more accurately and was significantly correlated with CuEXC. Longitudinal off-therapy 24 h-UCE values were theoretically expected, with significantly decreasing UCE values over the 12-month observation period, whereas 24 h-UCE sampling without therapy interruption did not. This study is the first to obtain real-world data from pairwise measurements, showing that the discrepancy between the two sampling methods for 24 h-UCE in WD patients can falsely influence dose adjustments during maintenance therapy in approximately 50% of patients.

## Electronic supplementary material

Below is the link to the electronic supplementary material.

Supplementary Material 1

Supplementary Material 2

## Data Availability

All data generated or analysed during this study are included in this published article and its supplementary information files. The datasets used and/or analysed during the current study are available from the corresponding author [I Mohr] on reasonable request.

## Abbreviations

WD: Wilson disease
DPA: D-penicillamine
24h-UCE: 24 h urinary copper excretion
NCC: nonceruloplasmin-bound copper
CuEXC: exchangeable copper
AASLD: American Association for the Study of Liver Diseases
EASL: European Association for the Study of the Liver
TETA1 HCl: trientine dihydrochloride
TETA4 HCl: trientine tetrahydrochloride
PET: positron emission tomography
