# Supplementary material for: A comparative analysis in monitoring 24-hour urinary copper in wilson disease: sampling on or off treatment?
Source: Orphanet J Rare Dis. 2025 Jan 21;20:33. doi: 10.1186/s13023-025-03545-2 (PMC11748325; doi:10.1186/s13023-025-03545-2)
Supplement: Supplementary file 1 — Supplementary Material 1 [file 13023_2025_3545_MOESM1_ESM.docx]

**Therapy monitoring in Wilson Disease**

**24-hour urinary copper excretion within reference value 3-8µmol/d**

**51% 66% 56%**


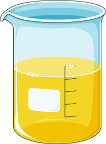

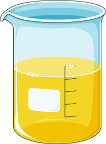

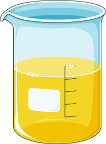


**Results in about half of the pairwise 24-hour urinary results would implicate discordant therapeutic conclusions**

**Sampling on**

**chelation therapy**

**No significant correlation with exchangable copper**

**Discordant 24-hour urinary copper results related to reference values**

**51% 41% 54%**

**24-hour**

**urinary**

**sample**


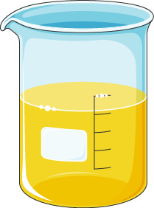


**Positive significant correlation with exchangable copper**


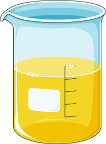

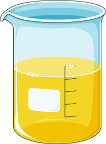

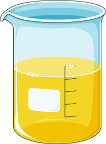


**24-hour urinary copper excretion within reference value < 1.6 µmol/d**

**47% 61% 47%**

**Sampling off**

**chelation therapy**
