# Supplementary material for: A comparative analysis in monitoring 24-hour urinary copper in wilson disease: sampling on or off treatment?
Source: Orphanet J Rare Dis. 2025 Jan 21;20:33. doi: 10.1186/s13023-025-03545-2 (PMC11748325; doi:10.1186/s13023-025-03545-2)
Supplement: Supplementary file 2 — Supplementary Material 2 [file 13023_2025_3545_MOESM2_ESM.docx]

**Supplements:**

**Supplemental table 1a: Descriptive statistics for key clinical and laboratory parameters in DPA-treated subgroup**

*Supplemental Table 1a depicts longitudinal development of DPA-dosage, 24h-UCE on- and off-therapy and additional markers of copper metabolism for patients under DPA.*

| Parameter | T0 | | T1 | | T2 | | T0 vs. T2 |
| --- | --- | --- | --- | --- | --- | --- | --- |
|  | n | Median (range; SD) | n*^1^ | Median (range; SD) | n*^1^ | Median (range; SD) | p-value*^2^ |
| DPA-dosage [mg/d] | 42 | 1153 (300-1800; 330) | 33 | 1215 (600-1800; 287) | 33 | 1159 (600 -1800; 311) | 0.40 |
| Weight adjusted dosage [mg DPA/kg body weight] | 42 | 16.0 (4.9 -21.4; 4.0) | 33 | 16.0 (6.7-22.2; 3.7) | 33 | 16.0 (8.3-21.8; 3.7) | 0.35 |
| 24h-UCE [µmol/d] after  48h dose interruption | 42 | 1.5 (0.2-5.3; 1.2) | 32 | 1.0 (0.2-3.9; 0.9) | 22 | 0.9 (0.4-4.0; 0.9) | 0.12 |
| 24h-UCE [µmol/d]  without dose interruption | 42 | 8.3 (2.4-27.4; 5.5) | 32 | 7.7 (2.6-26.2; 7.2) | 21 | 8.5 (2.0 -24.2; 5.6) | 0.26 |
| Serum copper [µmol/L] | 42 | 6.1 (1.3 -20; 4.1) | 30 | 6.2 (0.9 -16.3; 4.0) | 30 | 6.2 (0.9 -16.3; 4.0) | 0.88 |
| Coeruloplasmin [g/L] | 42 | 0.15 (0.03 -8.0; 1.4) | 28 | 0.12 (0.03-0.32; 0.1) | 28 | 0.12 (0.03 -0.32; 0.1) | 0.34 |
| NCC [g/L] (calculated) | 42 | 1.0 (0.02 -4.8; 1.1) | 17 | 1.1 (0.1 -3.3; 1.0) | 17 | 1.1 (0.1- 3.3; 1.0) | 0.63 |

Clinical presentation of WD un subgroup DPA: hepatic only n=27, neurologic only n=4, mixed presentation n=10

*^1^ data was not available for all patients at all times

*^2^ p-value was obtained using paired sample t-test, only paired values were included in the statistical calculation; p-palue <0.05 was regarded as statistically significant.

**Supplemental table 1b: Descriptive statistics for clinical and key laboratory parameters in Trientine-treated subgroup**

*Supplemental Table 1b depicts longitudinal development of trientine-dosage, 24h-UCE on- and off-therapy and additional markers of copper metabolism for patients under trientine.*

| Parameter | T0 | | T1 | | T2 | | T0 vs. T2 |
| --- | --- | --- | --- | --- | --- | --- | --- |
|  | n | Median (Min-Max; sd) | n*^1^ | Median (Min-Max; sd) | n*^1^ | Median (Min-Max; sd) | p-value*^2^ |
| Trientine-dosage [mg/d] | 42 | 877 (400-1200; 191) | 35 | 877 (400-1200; 197) | 38 | 902 (500-1400; 2.1) | 0.19 |
| Weight adjusted dosage [mg trientine/kg body weight] | 42 | 11.5 (5.3-19.2; 3.1) | 35 | 11.8 (5.3-19.6; 3.5) | 38 | 12.0 (6.9-20.0; 3.6) | 0.10 |
| 24h Urinary copper [µmol/d] after  48h dose interruption | 42 | 1.7 (0.4-5.2; 1.4) | 35 | 1.6 (0.3-4.7; 1.0) | 23 | 1.8 (0.4-3.6; 0.9) | 0.20 |
| 24h Urinary copper [µmol/d] without  dose interruption | 42 | 4.4 (0.8-15.6; 3.0) | 35 | 5.2 (1.5-16.8; 3.6) | 23 | 6.6 /2.5 -10.5; 2.6) | 0.87 |
| Serum copper [µmol/l] | 42 | 6.5 (1.4- 18.7; 4.0) | 35 | 5.9 (1.2-14.8: 3.7) | 38 | 5.7 (1.5-12.4; 3.3) | 0.34 |
| Coeruloplasmin [g/l] | 42 | 0.16 (0.03 -0.26; 0.1) | 35 | 0.17 (0.02 -0.3; 0.1) | 38 | 0.16 (0.02-0.2; 0.1) | 0.10 |
| NCC [g/L] (calculated) | 42 | 1.3 (0.01-3.8; 0.8) | 20 | 1.3 (0.2-3.5; 1.3) | 18 | 1.2 (1.2-3.0; 0.8) | 0.67 |

Clinical presentation of WD un subgroup Trientine: hepatic only n=28, mixed presentation n=15

*^1^ data was not available for all patients at all times

*^2^ p-value was obtained using paired sample t-test, only paired values were included in the statistical calculation

**Legend Table 1a and 1b:**

NCC= non-coeruloplasmin bound copper; SD= standard deviation, n= number

**Supplemental table 2: Correlation between 24h-UCE with and without 48h therapy interruption and NCC and CuEXC**

*Supplemental table 2 shows the different correlations of 24h-UCE on- and off-therapy at T0, T1 and T2 with NCC and CuEXC.*

|  |  | Timepoint matched 24h-UCE [µmol/d] **without**  dose interruption | NCC [g/l] (calculated) | CuEXC [µmol/l] |
| --- | --- | --- | --- | --- |
| T0 | 24h-UCE [µmol/d] with  48h dose interruption | p=0.198  Pearson Coefficient 0.143 | p=0.459  Pearson Coefficient 0.152 | **p=0.018***  Pearson Coefficient 0.260 |
|  | 24h-UCE [µmol/d] **without**  dose interruption | 1 | p=0.955  Pearson Coefficient 0.012 | p=0.752  Pearson Coefficient -0.036 |
| T1 | 24h-UCE [µmol/d] with  48h dose interruption | p=0.317  Pearson Coefficient 0,135 | p=0.943  Pearson Coefficient -0.013 |  |
|  | 24h-UCE [µmol/d] **without**  dose interruption | 1 | p=0.690  Pearson Coefficient -0.071 |  |
| T2 | 24h-UCE [µmol/d] with  48h dose interruption | p=0.444  Pearson Coefficient -0.121 | p=0.543  Pearson Coefficient -0.118 |  |
|  | 24h-UCE [µmol/d] **without**  dose interruption | 1 | p=0.465  Pearson Coefficient -0.139 |  |

**Legend table 2:**

NCC= non-coeruloplasmin bound copper; CuEXC= exchangeable copper, SD= standard deviation, *= significant

**Supplemental figure 1a and 1b: Patient individual 24h-UCE trajectories at T0, T1 und T2**

*Supplemental figure 1a and 1b show the 24h-UCE as graphical trajectoris from T0 to T2 without treatment interruption (supplemental figure 1a) and with treatment interruption (supplemental figure 1b).*

**Supplemental figure 2a: Scatterplot of T1 measurements of 24h UCE with and without treatment interruption**

*Supplemental figure 2a and 2b show 24h-UCE measurements at T1 (supplemental figure 2a) and at T2 (supplemental figure 2b) with and without treatment interruption. Guideline reference values are drawn with blue lines (EASL off therapy <1.6µmol/D; AASLD on therapy 3-8µmol/d). Green box shows concordant measurements, whereas red boxes reveal discordant values. Additionally, percentage of measurement of each box (A to F) is given in %.*

**D 1%**

**C 10%**

**B 25%**

**E 5%**

**F 34%**

**A 25%**

**Supplemental figure 2b: Scatterplot of T2 measurements of 24h UCE with and without treatment interruption**

**D 2%**

**C 16%**

**E 9%**

**B 24%**

**F 22%**

**A 27%**

Legend: = discordant pairwise UCE; concordant pairwise UCE;

= Guidelines reference values (EASL: UCE off therapy <1.6µmol/D; AASLD: UCE on therapy 3-8µmol/d)

= UCE pairwise measurements

***Supplemental information on Methods***

1. ***24h-urinary-sampling***

Urinary sampling over 24h und measuring urinary copper excretion (UCE) was done pairwise at each visit timepoint (T0, T1 and T2). Pairwise urinary copper sampling was defined as 24h-urinary-sampling after 48h of treatment interruption 14 days prior to follow-up appointment (FU) and 24h-urinary-sampling without treatment interruption the day prior to FU appointment. The method of 24h-urinary-sampling is explained in the following: The collection period begins in the morning after waking up. The patient was instructed to empty bladder in the morning as usual. This first morning urine is not collected. The time of urination is noted, as from there on the collection period starts. For the following 24 hours, all urine portions, including nighttime, are collected in the urine collection container, including the bladder emptying directly after waking up the next morning. The urine volume is measured and recorded. Subsequently, the collected urine is mixed. To do this, the urine collection container is swirled to ensure thorough mixing. If more than one urine collection container is used, the contents of all containers are first poured into a clean bucket. Then a sample is transferred from the collection container into a tube for analysis.

1. ***Exchangeable copper (CuEXC) Measurement***

For CuEXC determination, blood samples (serum) were collected in S-Monovette® Serum CAT 7,5ml (Sarstedt) and instantaneously transferred to our laboratory to be treated within 30 min. Blood was centrifuged at 1200G for 10 min and serum was prepared immediately for ultrafiltration. A two-step method (ultrafiltration-determination) was carried out to determine CuEXC by using EDTA as a chelator of high-copper-affinity. Sample extraction: To 1 ml of serum, add 1 ml of extraction solution (3.015 g of Titriplex III dissolved in 1000 ml of 0.9% NaCl solution). Subsequently, vortex the sample for 20 seconds. After an incubation period of 1 hour, transfer the sample to an ultracentrifugal filter (Amicon-Ultra-4, 30 kDa). After centrifugation for 20 minutes at 1200 G (3000 RPM with a 12 cm radius centrifuge), remove the filter and determine the copper content using the formula REC (%) = CuEXC [µmol/l]/ total serum copper [µmol/l] x 100. The measurements of copper in ultrafiltrates were performed by Zeeman AA 240 Z graphite furnace atomic absorption spectrometry. 40 μL of sample was injected into the furnace. Calibration for copper is performed with a standard solution (0.47 µmol/l), from which the device automatically creates two further dilutions (1:1.43 (0.329 µmol/l) and 1:3.3 (0.141 µmol/l)). Calibration is done at the beginning of each measurement series. The zero point (CAL ZERO) is determined at the start of calibration using a blank solution (extinction ≤ 0.02). The blank solution and the copper standards 1 – 4 are measured in triplicate, and the results are averaged. Calibration is calculated from these averages. The deviations of the triplicate standards must not exceed 20%. External calibration was conducted for copper at 327.4 nm.
